# Supplementary figures and images for: Primary care quality for older adults: Practice-based quality measures derived from a RAND/UCLA appropriateness method study
Source: PLoS One. 2024 Jan 19;19(1):e0297505. doi: 10.1371/journal.pone.0297505 (PMC10798529; doi:10.1371/journal.pone.0297505)

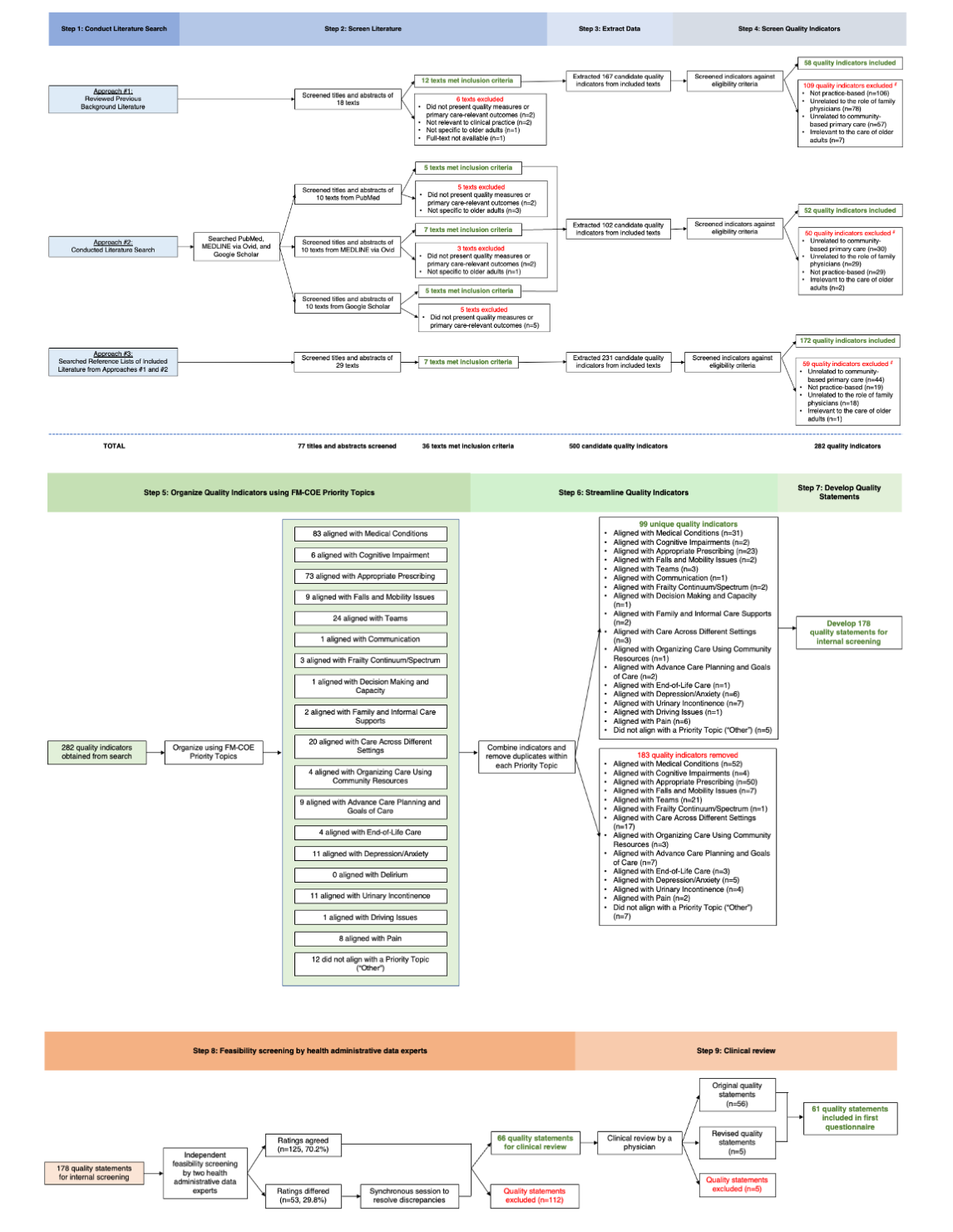

Supplement: S1 Fig — Legend: Steps 1 to 4 outline the literature search results of 282 candidate indicators. Steps 5 to 7 illustrate how the candidate indicators were organized by the FM-COE Priority Topics and streamlined into 178 quality statements. Steps 8 and 9 display the results of internal feasibility screening and clinical review, resulting in 61 quality statements for inclusion in the first questionnaire. (TIFF) [file pone.0297505.s001.tiff]

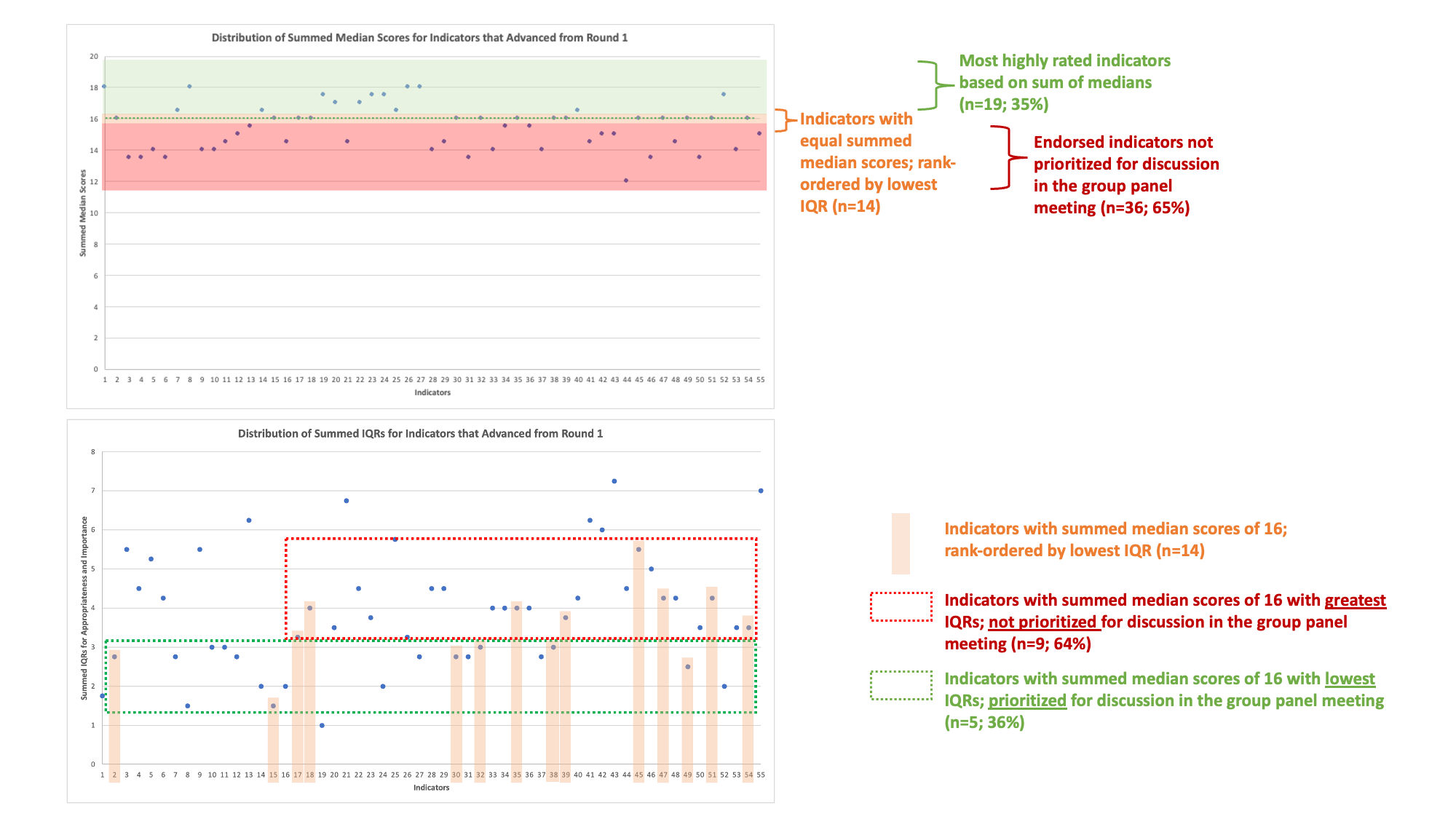

Supplement: S2 Fig — Legend: 19 indicators whose summed median scores were rated most highly were prioritized for discussion in the group panel meeting. 14 indicators had equal summed median scores and were rank-ordered by the lowest summed IQR for prioritization. (TIFF) [file pone.0297505.s002.tiff]

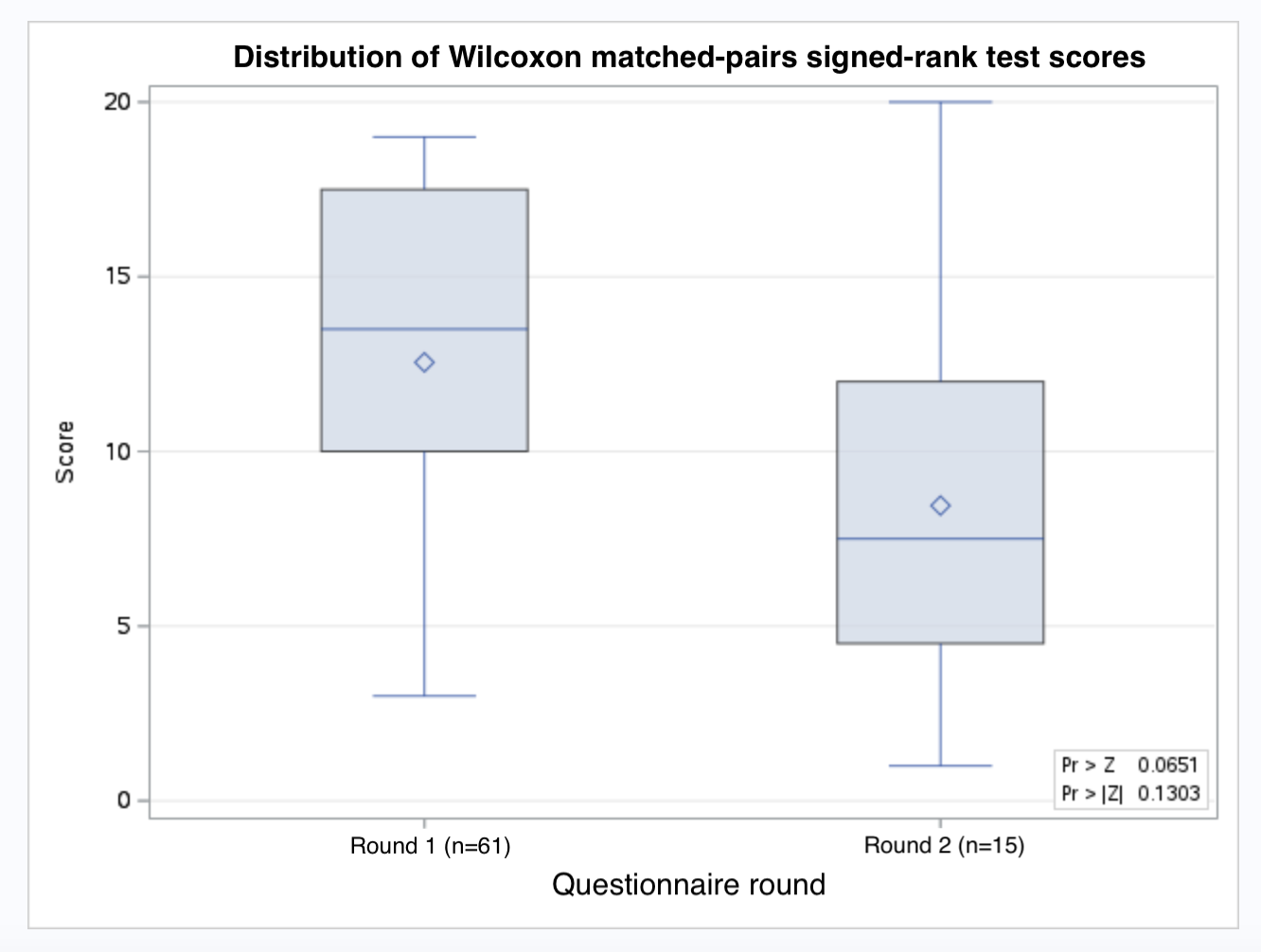

Supplement: S3 Fig — Legend: The p-value obtained by the Wilcoxon matched-pairs signed-rank test suggests a significant difference in consensus scores between the two RAM rounds. (TIFF) [file pone.0297505.s003.tiff]
