# Supplementary material for: Primary care quality for older adults: Practice-based quality measures derived from a RAND/UCLA appropriateness method study
Source: PLoS One. 2024 Jan 19;19(1):e0297505. doi: 10.1371/journal.pone.0297505 (PMC10798529; doi:10.1371/journal.pone.0297505)
Supplement: S2 Table — (DOCX) [file pone.0297505.s005.docx]

**S2 Table. Summary of included literature**

| **Reference** | | **Setting** | **Aim / Purpose** | **Literature Type** | **Source** |
| --- | --- | --- | --- | --- | --- |
| 1 | Horgan, S., Kay, K., & Morrison, A. (2020, August). Designing Integrated Care for Older Adults Living with Complex and Chronic Health Needs: A Scoping Review. Provincial Geriatrics Leadership Office. | Canada | Identify the core design elements that guide integrated care for older persons, and examine how best-practice models operationalize these design elements in practice. | Report (Scoping review) | <https://rgps.on.ca/wp-content/uploads/2020/11/PGLO-Scoping-Review-FINAL.pdf> |
| 2 | Akpan, A., Roberts, C., Bandeen-Roche, K., Batty, B., Bausewein, C., Bell, D., ... & Banerjee, J. (2018). Standard set of health outcome measures for older persons. BMC Geriatrics, 18(1), 1-10. | Multi-national | Develop multiple global outcome measures in older persons to support the ability of healthcare systems to improve their care pathways and quality of care. | Peer-reviewed article (Modified Delphi study) | doi: 10.1186/s12877-017-0701-3 |
| 3 | INSPIRE-PHC. (2023). Primary Care Data Reports for Ontario Health Teams. | Canada | Use standardized health administrative measures in primary care to provide a deeper understanding of the attributed population of each Ontario Health Team. | Data report | <https://www.ontariohealthprofiles.ca/ontarioHealthTeam.php#OHT_map> |
| 4 | World Health Organization. (2019). Integrated care for older people (ICOPE) implementation framework: Guidance for systems and services. Geneva. Licence: CC BY-NC-SA 3.0 IGO | Geneva | Describe the Integrated Care for Older People (ICOPE) Implementation Framework to assess and measure the capacity of services and systems to deliver integrated care at the community-level. | Report | <https://www.who.int/publications/i/item/9789241515993> |
| 5 | World Health Organization and the United Nations Children’s Fund (UNICEF). (2022). Web Annex. Technical specifications. In: Primary health care measurement framework and indicators: monitoring health systems through a primary health care lens. Geneva. Licence: CC BY-NC-SA 3.0 IGO. | Geneva | Provide technical specifications for each indicator included in the menu of indicators proposed for primary health care measurement framework and indicators. | Report | <https://apps.who.int/iris/handle/10665/352201> |
| 6 | Canadian Institute for Health Information. (2022). Common Challenges, Shared Priorities: Measuring Access to Home and Community Care and to Mental Health and Substance Use Services in Canada — Volume 4. Ottawa, ON. | Canada | Describe the development and reporting of the Shared Health Priorities indicators that were endorsed by the federal, provincial, and territorial governments in 2017. | Report | <https://www.cihi.ca/sites/default/files/document/common-challenges-shared-priorities-vol-4-report-en.pdf> |
| 7 | Canadian Institute for Health Information. (2023). Indicator Library. | Canada | Provides definitions and methodologies for more than 100 CIHI indicators, ranging from acute care, continuing care and rehabilitation to finance, spending and health human resources. | Indicator library | <https://secure.cihi.ca/free_products/Pan-Canadian_PHC_Indicator_Update_Report_en_web.pdf> |
| 8 | Health Quality Ontario. (2023). Indicator Library. | Canada | Provides a fully searchable library of indicator profiles reported by HQO and other entities. | Indicator library | <http://indicatorlibrary.hqontario.ca/Indicator/Search/EN> |
| 9 | Ministry of Health and Long-Term Care. (2014). Assessment & Restore Guideline. | Canada | Define the elements of the Assess & Restore (A&R) approach to care and set out the Ministry’s expectations with respect to the planning, establishment, delivery, monitoring, and evaluation of A&R initiatives. | Report | <https://www.health.gov.on.ca/en/pro/programs/assessrestore/docs/ar_guideline.pdf> |
| 10 | Health Canada. (2013). Healthy Canadians 2012: A Federal Report on Comparable Health Indicators. Ottawa. | Canada | Reports on the health status of Canadian and the performance of the health care system based on 53 indicators. | Report | <https://www.canada.ca/en/health-canada/services/health-care-system/reports-publications/health-care-system/healthy-canadians-federal-report-comparable-health-indicators-2012.html> |
| 11 | Regional Geriatric Program of Toronto. (2017). The Senior Friendly Care Framework. | Canada | Introduce the Senior Friendly Care Framework and provide highlights of the development process. | Report | <https://www.rgptoronto.ca/wp-content/uploads/2017/12/sfCare_Framework.pdf> |
| 12 | Kromm S, Mondor L, Wodchis WP. (2015). Assessing Value in Ontario Health Links. Part 3: Measures of System Performance in Ontario’s Health Links. Health System Performance Research Network. Volume 4. Toronto. | Canada | Describe the characteristics of the population in Health Links regions; measure health system performance in HL regions using data held at the Institute for Clinical Evaluative Sciences (ICES), creating a portrait of HLs that can be used in the future; and compare system performance among HLs and to existing physician networks (PN), defined by referral patterns among primary care physicians. | Report | <https://hspn.ca/wp-content/uploads/2019/09/HSPRN-AHRQ-Health-Links-Part-3-Measures.pdf> |
| 13 | Thyrian JR, Hertel J, Wucherer D, Eichler T, Michalowsky B, Dreier-Wolfgramm A, Zwingmann I, Kilimann I, Teipel S, Hoffmann W. (2017). Effectiveness and Safety of Dementia Care Management in Primary Care: A Randomized Clinical Trial. JAMA Psychiatry, 1;74(10):996-1004. | Canada | Test the effectiveness and safety of Dementia Care Management in the treatment and care of people with dementia living at home and caregiver burden. | Peer-reviewed article (Randomized clinical trial) | doi: 10.1001/jamapsychiatry.2017.2124 |
| 14 | Pottie, K., Thompson, W., Davies, S., Grenier, J., Sadowski, C. A., Welch, V., ... & Farrell, B. (2018). Deprescribing benzodiazepine receptor agonists: Evidence-based clinical practice guideline. *Canadian Family Physician*, *64*(5), 339-351. | Canada | Develop an evidence-based guideline to help clinicians make decisions about when and how to safely taper and stop benzodiazepine receptor agonists (BZRAs); to focus on the highest level of evidence available and seek input from primary care professionals in the guideline development, review, and endorsement processes. | Peer-reviewed article (Clinical guideline development) | PMID: 29760253 |
| 15 | Kim, K. I., Jung, H. K., Kim, C. O., Kim, S. K., Cho, H. H., Kim, D. Y., Ha, Y. C., Hwang, S. H., Won, C. W., Lim, J. Y., Kim, H. J., Kim, J. G., & Korean Association of Internal Medicine, The Korean Geriatrics Society. (2017). Evidence-based guidelines for fall prevention in Korea. The Korean Journal of Internal Medicine, 32(1), 199–210. | Korea | Develop guidelines and provide detailed recommendations and concrete measures to assess the risk of falls and prevent falls among older people. | Peer-reviewed article (Clinical guideline development) | doi: 10.3904/kjim.2016.218 |
| 16 | Romskaug, R., Skovlund, E., Straand, J., Molden, E., Kersten, H., Pitkala, K. H., ... & Wyller, T. B. (2020). Effect of clinical geriatric assessments and collaborative medication reviews by geriatrician and family physician for improving health-related quality of life in home-dwelling older patients receiving polypharmacy: a cluster randomized clinical trial. JAMA Internal Medicine, 180(2), 181-189. | Norway | Investigate the effect of clinical geriatric assessments and collaborative medication reviews by geriatrician and family physician on health-related quality of life and other patient-relevant outcomes in home-dwelling older patients receiving polypharmacy. | Peer-reviewed article (Randomized clinical trial) | doi: 10.1001/jamainternmed.2019.5096 |
| 17 | Mays, A. M., Saliba, D., Feldman, S., Smalbrugge, M., Hertogh, C. M., Booker, T. L., ... & Katz, P. R. (2018). Quality indicators of primary care provider engagement in nursing home care. Journal of the American Medical Directors Association, 19(10), 824-832. | Multi-national | Identify quality indicators germane to the international practice of primary care providers in post-acute and long-term care in order to demonstrate the added value of medical providers in nursing homes. | Peer-reviewed article (Modified Delphi study) | doi: 10.1016/j.jamda.2018.08.001 |
| 18 | Chadborn, N. H., Devi, R., Williams, C., Sartain, K., Goodman, C., Gordon, A. L. (2021). GPs' involvement to improve care quality in care homes in the UK: a realist review. | United Kingdom | Review reports of research and quality improvement (or similar change management) in care homes to explore how general practitioners have been involved; and develop programme theories explaining the role of general practitioners in improvement initiatives and outcomes. | Peer-reviewed article (Realist review) | doi: 10.3310/hsdr09200 |
| 19 | Snooks H, Bailey-Jones K, Burge-Jones D, et al. (2018). Predictive risk stratification model: a randomised stepped-wedge trial in primary care (PRISMATIC). Southampton (UK): NIHR Journals Library. | United Kingdom | Evaluate the introduction of predictive risk stratification in primary care. | Peer-reviewed article (Randomized clinical trial) | doi: 10.3310/hsdr06010 |
| 20 | Bosch-Lenders, D., Jansen, J., Stoffers, Winkens, B., Aretz, K., Twellaar, M., Schols, J. M. G. A., van der Kuy, P. M., Knottnerus, J. A., van den Akker, M. (2021). The effect of a comprehensive, interdisciplinary medication review on quality of life and medication use in community dwelling older people with polypharmacy. Journal of Clinical Medicine, 10(4). | Netherlands | Conduct a comprehensive medication review at the patients’ home and evaluate the effect of this medication review on quality of life and medication use. | Peer-reviewed article (Randomized clinical trial) | doi: 10.3390/jcm10040600 |
| 21 | Ekwegh, U., Dean, J. (2020). Improving care planning and communication for frail older persons across the primary-secondary care interface. Future Healthcare Journal, 7(3), e23-e26. | United Kingdom | Develop effective handover communication between the frailty team and primary care for patients assessed and transferred home from an emergency department. | Peer-reviewed article (Plan, do, study, act) | doi: 10.7861/fhj.2019-0052 |
| 22 | Dyer, S. M., Suen, J., Williams, H., Inacio, M. C., Harvey, G., Roder, D., Wesselingh, S., Kellie, A., Crotty, M., Caughey, G. E. (2022). Impact of relational continuity of primary care in aged care: a systematic review. BMC Geriatrics, 22(1), 579. | Australia | Examine the impact of relational continuity between primary care professionals and older people receiving aged care services, in residential or home care settings, on health care resource use and person-centred outcomes. | Peer-reviewed article (Systematic review) | doi: 10.1186/s12877-022-03131-2 |
| 23 | Hetlevik, O., Holmas, T. H., Monstad, K. (2021). Continuity of care, measurement and association with hospital admission and mortality: a registry-based longitudinal cohort study. BMJ Open, 11(12), e051958. | Norway | Assess whether continuity of care (COC) with a general practitioner (GP) is associated with mortality and hospital admissions for older patients. | Peer-reviewed article (Cohort study) | doi: 10.1136/bmjopen-2021-051958 |
| 24 | Mahlknecht, A., Wiedermann, C. J., Sandri, M., Engl, A., Valentini, M., Vogele, A., Schmid, S., Deflorian, F., Montalbano, C., Koper, D., Bellmann, R., Sonnichsen, A., Piccoliori, G. (2021). Expert-based medication reviews to reduce polypharmacy in older patients in primary care: a northern-Italian cluster-randomised controlled trial. BMC Geriatrics, 21(1), 659. | Italy | Achieve clinical benefits for older patients (aged 75+) by means of evidence-based reduction of polypharmacy (defined as ≥8 prescribed drugs) and inappropriate prescribing in general practice. | Peer-reviewed article (Randomized clinical trial) | doi: 10.1186/s12877-021-02612-0 |
| 25 | Kurtzman, E. T., & Barnow, B. S. (2017). A comparison of nurse practitioners, physician assistants, and primary care physicians’ patterns of practice and quality of care in health centers. Medical Care, 55(6), 615-622. | United States | To compare the quality of care and practice patterns of nurse practitioners, physician assistants, and primary care physicians in community health centres. | Peer-reviewed article (Cohort study) | <https://www.jstor.org/stable/26418382> |
| 26 | Hogg, W., Lemelin, J., Dahrouge, S., Liddy, C., Armstrong, C. D., Legault, F., ... & Zhang, W. (2009). Randomized controlled trial of anticipatory and preventive multidisciplinary team care: for complex patients in a community-based primary care setting. Canadian family physician, 55(12), e76-e85. | Canada | Examine whether quality of care improves when nurse practitioners and pharmacists work with family physicians in community practice and focus their work on patients who are 50 years of age and older and considered to be at risk of experiencing adverse health outcomes. | Peer-reviewed article (Randomized clinical trial) | <https://www.cfp.ca/content/cfp/55/12/e76.full.pdf> |
| 27 | Chen, L. M., Farwell, W. R., & Jha, A. K. (2009). Primary care visit duration and quality: does good care take longer?. Archives of internal medicine, 169(20), 1866-1872. | United States | Describe changes in the duration of adult primary care visits and in the quality of care provided during these visits and to determine whether quality of care is associated with visit duration. | Peer-reviewed article (Cohort study) | doi: 10.1001/archinternmed.2009.341 |
| 28 | Terrell, K. M., Hustey, F. M., Hwang, U., Gerson, L. W., Wenger, N. S., Miller, D. K., & Society for Academic Emergency Medicine (SAEM) Geriatric Task Force. (2009). Quality indicators for geriatric emergency care. Academic Emergency Medicine, 16(5), 441-449. | United States | Develop emergency department-specific quality indicators for older patients to help practitioners identify quality gaps and focus quality improvement efforts. | Peer-reviewed article (Consensus study) | doi: 10.1111/j.1553-2712.2009.00382.x |
| 29 | Leff, B., Carlson, C. M., Saliba, D., & Ritchie, C. (2015). The invisible homebound: setting quality-of-care standards for home-based primary and palliative care. Health Affairs, 34(1), 21-29. | United States | Describe the current status of home-based medical care in the United States and the network’s quality-of-care framework. | Peer-reviewed article (Literature synthesis) | doi: 10.1377/hlthaff.2014.1008 |
| 30 | Heckman, G. A., Hillier, L., Manderson, B., McKinnon-Wilson, J., Santi, S. M., & Stolee, P. (2013). Developing an integrated system of care for frail seniors. Healthcare Management Forum, 26(4), 200-208. SAGE Publications. | Canada | Undertake a consultation process with healthcare providers to assess current system strengths, challenges and gaps in providing care to frail seniors. | Peer-reviewed article (Qualitative study) | doi: 10.1016/j.hcmf.2013.09.003 |
| 31 | Frank, C., & Wilson, C. R. (2015). Models of primary care for frail patients. Canadian Family Physician, 61(7), 601-606. | Canada | Discuss models of care for frail seniors provided in primary care settings and those developed by Canadian family physicians. | Peer-reviewed article (Literature review) | PMCID: PMC4501602 |
| 32 | Higashi, T., Shekelle, P. G., Adams, J. L., Kamberg, C. J., Roth, C. P., Solomon, D. H., ... & Wenger, N. S. (2005). Quality of care is associated with survival in vulnerable older patients. Annals of Internal Medicine, 143(4), 274-281. | United States | Examine the link between the quality of care that patients received and their survival. | Peer-reviewed article (Cohort study) | doi: 10.7326/0003-4819-143-4-200508160-00008 |
| 33 | Canadian Geriatrics Society. (2022). Eight tests and treatments to question. Choosing Wisely Canada. | Canada | Ensure recommendations and background information from the American Geriatrics Society were valid and relevant for Canadian patients and our health care system. | Web page | <https://choosingwiselycanada.org/recommendation/geriatrics/> |
| 34 | AMDA. (2022). Fifteen Things Physicians and Patients Should Question. Choosing Wisely. | United States | Achieve consensus on clinical recommendations relevant to the quality of care for long-term care patients. | Web page | <https://www.choosingwisely.org/societies/amda-the-society-for-post-acute-and-long-term-care-medicine/> |
| 35 | AGS Choosing Wisely Workgroup. (2014). American Geriatrics Society identifies another five things that healthcare providers and patients should question. Journal of the American Geriatrics Society, 62(5), 950-960. | United States | Engage healthcare organizations and professionals, individuals, and family caregivers in discussions about the safety and appropriateness of medical tests, medications, and procedures to identify five tests, medications, or procedures that appear to harm rather than help. | Peer-reviewed article (Clinical guideline development) | doi: 10.1111/jgs.12770 |
| 36 | Muth, C., Blom, J. W., Smith, S. M., Johnell, K., Gonzalez‐Gonzalez, A. I., Nguyen, T. S., ... & Valderas, J. M. (2019). Evidence supporting the best clinical management of patients with multimorbidity and polypharmacy: a systematic guideline review and expert consensus. Journal of Internal Medicine, 285(3), 272-288. | Multi-national | Identify and analyse available evidence-based clinical practice guidelines for multimorbidity or polypharmacy in order to investigate the clinical decision support they provide and the key concepts they address. | Peer-reviewed article (Clinical guideline development) | doi: 10.1111/joim.12842 |
