# Supplementary material for: Primary care quality for older adults: Practice-based quality measures derived from a RAND/UCLA appropriateness method study
Source: PLoS One. 2024 Jan 19;19(1):e0297505. doi: 10.1371/journal.pone.0297505 (PMC10798529; doi:10.1371/journal.pone.0297505)
Supplement: S4 Table — (DOCX) [file pone.0297505.s007.docx]

**S4 Table. Example of a technical definition for an endorsed quality statement**

| **Quality Indicator #11** **ORIGINAL:** IF an older primary care patient with no history of allergy to the pneumococcal vaccine is not known to have already received a pneumococcal vaccine or if the patient received it more than 5 years ago (if before age 65 years), THEN a pneumococcal vaccine should be offered. **REVISED:** IF an older primary care patient is not known to have already received a pneumococcal vaccine or if the patient received it more than 5 years ago (if before age 65 years), THEN a pneumococcal vaccine should be administered. | |
| --- | --- |
| **Definition:** Proportion of attached patients who have received the pneumococcal vaccine.  **Interpretation:** A high rate for this indicator can be interpreted as better. | |
| **DENOMINATOR: IF** an older primary care patient is not known to have already received a pneumococcal vaccine or if the patient received it more than 5 years ago (if before age 65 years), | |
| **Definition** | **Data Sources** |
| Number of older patients who are eligible for the pneumococcal vaccine.  **Inclusions:**   - Patient is an Ontario resident eligible for OHIP, aged 65 years or older. - Patient is attached to the PCP.   **Exclusions:**   - None | 1. Patient appears in ***PCPOP***   **AND**   1. Patient’s age group in PCPOP: ***AGEGP = Any of 7, 8, or 9*** *where 7 = 65-74 years, 8 = 75-84 years, and 9 = 85+ years*   **Notes:**   - We cannot determine whether a patient is eligible for the vaccine (i.e., whether the patient has “no history of allergy”) in the absence of patient-level medical data at ICES (e.g., allergies captured in an EMR). Therefore, this condition has been removed from the denominator. |
| **NUMERATOR: THEN** a pneumococcal vaccine should be administered. | |
| **Definition** | **Data Sources** |
| Number of older patients who received the pneumococcal vaccination.  **Inclusions:**   - Attached patients who received at least one dose of the immunization after the age 65.   **Exclusions:**   - This indicator only reflects immunizations associated with a physician fee code (i.e., billing record). For example, if an immunization was provided by a nurse practitioner, pharmacist, or a provider from outside Ontario, the immunization record would only appear in OHIP if the patient informed their PCP and the PCP noted this in their medical record. | 1. ***OHIP*** pneumococcal vaccination fee code: ***G846*** *(Pneumococcal Conjugate)*   **Notes:**   - None |
| **Calculation:** (Numerator ÷ Denominator) x 100%  **References:**   1. Public Health Agency of Canada. Pneumococcal vaccine: Canadian Immunization Guide [Internet]. fitch. 2007 [cited 2023 Jun 6]. Available from: https://www.canada.ca/en/public-health/services/publications/healthy-living/canadian-immunization-guide-part-4-active-vaccines/page-16-pneumococcal-vaccine.html 2. NIA. As One of Canada’s Top Killers, Why Isn’t Pneumonia Taken More Seriously [Internet]. 2019 [cited 2023 Jun 12]. Available from: https://static1.squarespace.com/static/5c2fa7b03917eed9b5a436d8/t/6446e6d5efce9313c4316e25/1682368214300/Pneumonia_Report+-+Final.pdf | |
