# Supplementary material for: Primary care quality for older adults: Practice-based quality measures derived from a RAND/UCLA appropriateness method study
Source: PLoS One. 2024 Jan 19;19(1):e0297505. doi: 10.1371/journal.pone.0297505 (PMC10798529; doi:10.1371/journal.pone.0297505)
Supplement: S5 Table — (DOCX) [file pone.0297505.s008.docx]

**S5 Table. Prioritization of endorsed items from round 1 to round 2**

|  | **Quality Statement** | **Endorsed in questionnaire 1 but not presented in panel group meeting**  (n=36) | **Presented in panel group meeting**  (n=19) | **Rated in questionnaire 2**  (n=15) |
| --- | --- | --- | --- | --- |
| 1 | IF an older primary care patient presents with a health concern, THEN the PCP conducts a focused clinical assessment. |  | X | X |
| 2 | IF a comprehensive geriatric assessment is performed on an older primary care patient, THEN the PCP should follow-up to ensure the implementation of recommendations. |  | X | X |
| 3 | IF an older primary care patient receives a cataract diagnosis, THEN the PCP should assess their visual function with respect to carrying out needed or desired activities every 12 months. | X |  |  |
| 4 | IF an older primary care patient has diabetes, THEN the PCP should recommend an ACE inhibitor or ARB, measure HbA1C at least twice annually, and examine feet and eyes every 2 years. | X |  |  |
| 5 | IF an older primary care patient with diabetes does not have established renal disease and is not receiving an ACE inhibitor or ACE receptor blocker, THEN the PCP should test for proteinuria annually. | X |  |  |
| 6 | IF an older primary care patient with diabetes has a LDL >2.0, ApoB>0.8, or nonHDL-c >2.6 , THEN the PCP should offer an intervention to lower cholesterol. | X |  |  |
| 7 | IF an older primary care patient with no history of allergy to the pneumococcal vaccine is not known to have already received a pneumococcal vaccine or if the patient received it more than 5 years ago (if before age 65 years), THEN a pneumococcal vaccine should be offered. |  | X | X |
| 8 | IF an older primary care patient has no history of anaphylactic hypersensitivity to eggs or to other components of the influenza vaccine, THEN the patient should be offered an annual influenza vaccination. |  | X | X |
| 9 | IF an older primary care patient presents for care, THEN the patient should be offered the shingles vaccine. | X |  |  |
| 10 | IF an older female primary care patient receives a new diagnosis of osteoporosis, THEN the PCP should offer bisphosphonate/denosumab followed by estrogen receptor agonist (only in those with menopausal symptoms) within 3 months. | X |  |  |
| 12 | IF an older primary care patient has rheumatoid arthritis, THEN the PCP should conduct a face-to-face annual review every 12 months. | X |  |  |
| 13 | IF an older primary care patient is diagnosed with coronary artery disease, THEN the PCP should recommend aspirin, beta-blockers, and/or statins. | X |  |  |
| 13 | IF an older primary care patient has established coronary artery disease and their cholesterol level is not known, THEN the PCP should order a fasting cholesterol evaluation, including total LDL and HDL cholesterol. | X |  |  |
| 14 | IF an older primary care patient has chronic obstructive pulmonary disease, THEN the PCP should recommend influenza immunization, pneumococcal vaccination, and the use of bronchodilators. |  | X | X |
| 15 | IF an older primary care patient has congestive heart failure, THEN the PCP should order ACE inhibitors or ARB and beta-blockers. |  | X | X |
| 16 | IF an older primary care patient receives a new diagnosis of heart failure, THEN the PCP should offer an evaluation of LV ejection fraction within 1 month. | X |  |  |
| 17 | IF an older primary care patient receives a new diagnosis of heart failure, THEN the PCP should order the following studies within 1 month of the diagnosis (unless they have already been performed within the previous 3 months): chest radiography, electrocardiography, CBC, serum sodium and potassium levels, serum creatinine level, and TSH level in patients with AF or heart failure with no obvious cause. | X |  |  |
| 18 | IF an older primary care patient has an elevated glycated hemoglobin level, THEN the PCP should offer a therapeutic intervention aimed at improving glycemic control within 3 months if the glycated hemoglobin level is 9.0% to 10.9%, and within 1 month if the glycated hemoglobin level is 11% or more. | X |  |  |
| 19 | IF an older primary care patient is diagnosed with dementia, THEN the PCP should provide dementia care management. |  | X | X |
| 20 | IF an older primary care patient receives a new diagnosis of dementia, THEN the PCP should perform a serum vitamin B12 and TSH test. |  | X | X |
| 21 | IF an older primary care patient is discharged from hospital, THEN the PCP should conduct a medication reconciliation within 14 days. | X |  |  |
| 22 | IF an older primary care patient presents for care, THEN the PCP should perform and document a medication review and reconciliation at least once a year (i.e., evaluate the risk benefit of each drug, its possible interactions and adverse effects, adherence to treatment and unmet needs and be aware of possible prescribing cascades). |  | X |  |
| 23 | IF an older primary care patient is prescribed medications from multiple providers, THEN the PCP should conduct a collaborative medication review (e.g., focus on evidence-based new drug prescriptions and prevention of polypharmacy). |  | X | X |
| 24 | IF an older primary care patient requires medication, THEN the PCP should avoid prescribing potentially inappropriate medications (e.g., drugs from the Beers list) unless clearly documented why that medication should be prescribed. |  | X | X |
| 25 | IF an older primary care patient is prescribed a drug from the Beers list, THEN the PCP should monitor chronic use of drugs from the Beers List. |  | X |  |
| 26 | IF an older primary care patient requires a new medication, THEN the PCP should not prescribe a medication with strong anticholinergic effects if alternatives are available. |  | X | X |
| 27 | IF an older primary care patient has symptoms of insomnia, agitation or delirium, THEN the PCP should not use benzodiazepines or other sedative-hypnotics as the first choice. |  | X | X |
| 28 | IF an older primary care patient is prescribed BZRAs, THEN the PCP should deprescribe (taper), regardless of duration of use. | X |  |  |
| 29 | IF an older primary care patient is prescribed a new medication, THEN the PCP should conduct a drug regime review (medication reconciliation review) and consider opportunities for deprescribing. | X |  |  |
| 30 | IF an older primary care patient experiences behavioural and psychological symptoms of dementia, THEN the PCP should consider alternatives to antipsychotics as the first choice to treat. |  | X | X |
| 31 | IF an older primary care patient has advanced dementia, THEN the PCP should not routinely prescribe or continue acetylcholinesterase inhibitors or N-Methyl-D-Aspartate antagonists. | X |  |  |
| 32 | IF an older primary care patient with advanced dementia has been prescribed acetylcholinesterase inhibitors or N-Methyl-D-Aspartate antagonists, THEN the PCP should conduct periodic reassessments for perceived benefits (cognitive, functional, behavioural) and adverse effects, and consider deprescribing if the risks outweigh the benefits. | X |  |  |
| 33 | IF an older primary care patient is newly started on a diuretic, THEN the PCP should check serum potassium and creatinine levels within 1 month of the initiation of therapy. | X |  |  |
| 34 | IF an older primary care patient is prescribed a thiazide or loop diuretic, THEN the PCP should check electrolyte levels at least annually. | X |  |  |
| 35 | IF an older primary care patient is newly started on an ACE inhibitor, THEN the PCP should check serum potassium and creatinine levels within 1 month of the initiation of therapy. | X |  |  |
| 36 | IF an older primary care patient requires pharmacotherapy for treatment of hypertension, THEN the PCP should order a once- or twice-daily medication (unless there is documentation about the need for agents that require more frequent dosing). | X |  |  |
| 37 | IF an older primary care patient has hypertension and asthma, THEN the PCP should not use beta-blocker therapy for hypertension. | X |  |  |
| 38 | IF an older primary care patient remains hypertensive after nonpharmacologic intervention, THEN the PCP should initiate pharmacologic antihypertensive treatment. |  | X | X |
| 39 | IF a primary care patient is older than 75 years of age and/or has a history of peptic ulcer disease, gastrointestinal bleeding, or current warfarin use and the patient is being treated with a COX-2 nonselective NSAID, THEN the PCP should offer concomitant treatment with either a proton-pump inhibitor or H2 blocker (like famotidine). | X |  |  |
| 40 | IF an older primary care patient requires analgesia, THEN the PCP should not use meperidine. |  | X |  |
| 41 | IF an older primary care patient is prescribed warfarin, THEN the PCP should order an INR test at least every 6 weeks. | X |  |  |
| 42 | IF an older primary care patient is prescribed warfarin, THEN an INR should be determined within 4 days after initiation of therapy and at least every 6 weeks by the PCP. | X |  |  |
| 43 | IF an older primary care patient has established CHD and is not receiving warfarin, THEN the PCP should offer antiplatelet therapy. | X |  |  |
| 44 | IF an older primary care patient has high HbA1c levels, THEN the PCP should avoid using medications known to cause hyperglycemia to achieve HbA1c <7.5%. | X |  |  |
| 45 | IF an older primary care patient requires specialist consultation, THEN the PCP should collaborate with specialist services by providing referrals/access, follow-up, and monitoring. | X |  |  |
| 46 | IF an older primary care patient receives care from multiple providers, THEN the PCP should communicate with other providers to ensure continuity of care and coordination. | X |  |  |
| 47 | IF an older primary care patient is discharged from hospital, THEN the PCP should follow up with the patient within 14 days. | X |  |  |
| 48 | IF an older primary care patient seeks primary care services, THEN the patient receives continuous care from the same PCP in subsequent visits (beyond a specific episode of illness). | X |  |  |
| 49 | IF an older primary care patient could benefit from community-based supports/resources, THEN the PCP identifies and facilitates the appropriate use of these resources. |  | X |  |
| 50 | IF an older primary care patient receives a diagnosis of depression, THEN the PCP should offer antidepressant treatment or psychotherapy within 2 weeks (unless there is documentation within that period that the patient has improved or unless the patient has substance abuse or dependence). | X |  |  |
| 51 | IF an older primary care patient is started on an antidepressant medication, THEN the PCP should not use the following medications as first- or second-line therapy: tertiary amine tricyclics (amitriptyline, imipramine, doxepin, clomipramine, or trimipramine), monoamine oxidase inhibitors (unless atypical depression is present), benzodiazepines, or stimulants (except methylphenidate). | X |  |  |
| 52 | IF an older primary care patient receives a new diagnosis of moderate or severe dementia, THEN the PCP should advise the patient not to drive a motor vehicle, request that the Department of Motor Vehicles (or equivalent) retests the patient’s ability to drive, or refer the patient to a drivers’ safety or education course that includes assessment of driving ability consistent with provincial laws. |  | X | X |
| 53 | IF an older primary care patient is treated for a chronic painful condition, THEN the PCP should assess for a response within 6 months. | X |  |  |
| 54 | IF an older primary care patient requires after-hours primary care, THEN the patient should be able to access this care from their PCP or another provider. | X |  |  |
| 55 | IF an older primary care patient requires immediate health care services, THEN the patient should be able to access this care from their PCP or another provider. | X |  |  |
