# Supplementary material for: Primary care quality for older adults: Practice-based quality measures derived from a RAND/UCLA appropriateness method study
Source: PLoS One. 2024 Jan 19;19(1):e0297505. doi: 10.1371/journal.pone.0297505 (PMC10798529; doi:10.1371/journal.pone.0297505)
Supplement: S6 Table — (DOCX) [file pone.0297505.s009.docx]

**S6 Table. Summary of round 2 ratings**

|  | **Quality Statement** | **Appropriateness** | | **Importance** | |
| --- | --- | --- | --- | --- | --- |
|  |  | **Median** | **IQR** | **Median** | **IQR** |
| 1 | **ORIGINAL:** IF an older primary care patient has no history of anaphylactic hypersensitivity to eggs or to other components of the influenza vaccine, THEN the patient should be offered an annual influenza vaccination.  **REVISED:** IF an older primary care patient is eligible for the influenza vaccine, THEN the patient should be administered the vaccine annually. | 8.5 | 1 | 8 | 1 |
| 2 | **ORIGINAL:** IF an older primary care patient presents with a health concern, THEN the PCP conducts a focused clinical assessment.  **REVISED:** IF an older primary care patient presents for care, THEN the PCP conducts a focused clinical assessment. | 7 | 3.5 | 6.5 | 3.5 |
| 3 | **ORIGINAL:** IF an older primary care patient has symptoms of insomnia, agitation or delirium, THEN the PCP should not use benzodiazepines or other sedative-hypnotics as the first choice.  **REVISED:** IF an older primary care patient requires a new medication, THEN the PCP should not use benzodiazepines or other sedative-hypnotics as the first choice. | 8 | 1.5 | 8 | 1.5 |
| 4 | IF an older primary care patient requires a new medication, THEN the PCP should not prescribe a medication with strong anticholinergic effects if alternatives are available. | 8 | 1.75 | 8 | 1.75 |
| 5 | IF an older primary care patient is diagnosed with dementia, THEN the PCP should provide dementia care management. | 7.5 | 1.75 | 7 | 1 |
| 6 | **ORIGINAL:** IF an older primary care patient requires medication, THEN the PCP should avoid prescribing potentially inappropriate medications (e.g., drugs from the Beers list) unless clearly documented why that medication should be prescribed.  **REVISED:** IF an older primary care patient requires medication, THEN the PCP should avoid prescribing potentially inappropriate medications (e.g., drugs from the Beers list). | 8 | 1 | 7 | 2.75 |
| 7 | **ORIGINAL:** IF an older primary care patient receives a new diagnosis of moderate or severe dementia, THEN the PCP should advise the patient not to drive a motor vehicle, request that the Department of Motor Vehicles (or equivalent) retests the patient’s ability to drive, or refer the patient to a drivers’ safety or education course that includes assessment of driving ability consistent with provincial laws.  **REVISED:** IF an older primary care patient receives a new diagnosis of dementia and is deemed unsafe to drive, THEN the PCP should report the patient to the Ministry of Transportation. | 8.5 | 2.75 | 8 | 2.5 |
| 8 | IF an older primary care patient is prescribed medications from multiple providers, THEN the PCP should conduct a collaborative medication review (e.g., focus on evidence-based new drug prescriptions and prevention of polypharmacy). | 7.5 | 1.75 | 7.5 | 2.5 |
| 9 | **ORIGINAL:** IF an older primary care patient receives a new diagnosis of dementia, THEN the PCP should perform a serum vitamin B12 and TSH test.  **REVISED:** IF an older primary care patient presents with memory concerns, THEN the PCP should perform tests aligned with the 5th Canadian Consensus on Dementia. | 8 | 1.75 | 7.5 | 1 |
| 10 | **ORIGINAL:** IF an older primary care patient has chronic obstructive pulmonary disease, THEN the PCP should recommend influenza immunization, pneumococcal vaccination, and the use of bronchodilators.  **REVISED:** IF an older primary care patient has chronic obstructive pulmonary disease, THEN the PCP should recommend influenza and pneumococcal immunizations. | 9 | 1 | 8.5 | 1 |
| 11 | **ORIGINAL:** IF an older primary care patient with no history of allergy to the pneumococcal vaccine is not known to have already received a pneumococcal vaccine or if the patient received it more than 5 years ago (if before age 65 years), THEN a pneumococcal vaccine should be offered.  **REVISED:** IF an older primary care patient is not known to have already received a pneumococcal vaccine or if the patient received it more than 5 years ago (if before age 65 years), THEN a pneumococcal vaccine should be administered. | 8 | 1 | 8 | 0.75 |
| 12 | **ORIGINAL:** IF an older primary care patient has congestive heart failure, THEN the PCP should order ACE inhibitors or ARB and beta-blockers.  **REVISED:** IF an older primary care patient has congestive heart failure, THEN the PCP should order ACE inhibitors, ARBs, beta-blockers, or SGLT2 inhibitors. | 7.5 | 1 | 8 | 1.75 |
| 13 | **ORIGINAL:** IF an older primary care patient experiences behavioural and psychological symptoms of dementia, THEN the PCP should consider alternatives to antipsychotics as the first choice to treat.  **REVISED:** IF an older primary care patient is diagnosed with dementia, THEN the PCP should consider alternatives to antipsychotics as the first choice to treat. | 8 | 0.75 | 7.5 | 2 |
| 14 | IF a comprehensive geriatric assessment is performed on an older primary care patient, THEN the PCP should follow-up to ensure the implementation of recommendations. | 7 | 1.5 | 6.5 | 2 |
| 15 | **ORIGINAL:** IF an older primary care patient remains hypertensive after nonpharmacologic intervention, THEN the PCP should initiate pharmacologic antihypertensive treatment.  **REVISED:** IF an older primary care patient presents as hypertensive, THEN the PCP should initiate pharmacologic antihypertensive treatment. | 6 | 1.75 | 6.5 | 2 |
