# Supplementary material for: Primary care quality for older adults: Practice-based quality measures derived from a RAND/UCLA appropriateness method study
Source: PLoS One. 2024 Jan 19;19(1):e0297505. doi: 10.1371/journal.pone.0297505 (PMC10798529; doi:10.1371/journal.pone.0297505)
Supplement: Attachment — Submitted filename: plosone.docx [file pone.0297505.s010.docx]

Thank you for the opportunity to review this manuscript.

Using RAND/UCLA appropriateness study practice-based quality measures are derived to measure quality of care for older adults in primary care on population level

Overall, this is a well-written and interesting paper. The protocol paper has been published earlier.

Some questions and minor comments could enhance the readability of the paper.

Introduction

- Please clarify what you mean with ‘practice-based process metrics’
- An operationalization of quality of care Is not described, please elaborate how this concept is viewed by the authors.

Methods

- The steps are easy to follow. Hance please clarify the following aspects.
  - 1. Table 1 point 4 “practice-based orientation.

Results

- Round 1 questionnaire please describe the six indicators that were eliminated
- Many quotes from the panelists are described, what was done with this information?
- How many panelists were participating during each session

Discussion

- Please elaborate more why and how the selection was done to reduce the number of indicators – only time limitation is somewhat vague.
- Please elaborate on the generalizability of the available administrative data
