# Supplementary material for: Primary care quality for older adults: Practice-based quality measures derived from a RAND/UCLA appropriateness method study
Source: PLoS One. 2024 Jan 19;19(1):e0297505. doi: 10.1371/journal.pone.0297505 (PMC10798529; doi:10.1371/journal.pone.0297505)
Supplement: Attachment — Submitted filename: Response to reviewers_Jan 2024.docx [file pone.0297505.s012.docx]

**Rebecca Correia, PhD(c)**

**Department of Health Research Methods, Evidence, and Impact**

Faculty of Health Sciences

McMaster University

1280 Main Street West

Hamilton, ON, Canada L8N 3Z5

Email: correirh@mcmaster.ca

January 3, 2023

Mohammad Sadegh Taghizadeh
Academic Editor
PLOS ONE

RE:	PONE-D-23-32654R1– “Primary care quality for older adults: practice-based quality measures derived from a RAND/UCLA appropriateness method study”

Dear PLOS ONE Editorial Office and Reviewers:

We appreciate your continued support in strengthening our manuscript. Thank you for providing encouraging and constructive feedback. In our Response to Reviewers, we describe the changes made to our manuscript to address the outstanding peer review comments. All suggestions and comments from the reviewer have been incorporated.

Thank you again for your consideration.

Sincerely,

Rebecca Correia, BSc

PhD Candidate

Department of Health Research Methods, Evidence, and Impact, McMaster University

[correirh@mcmaster.ca](about:blank)

**Reviewer #1:**

This revision is improved, notably from a more robust discussion of limitations and methodological focus. The authors responded well to all reviewer comments.

There are remaining points/questions as below.

1. Line 28: I wouldn't say pan-Canadian. 9 were based in Ontario. Perhaps just Canadian

Thanks for this comment; we have revised accordingly.

Line 27-29:	We conducted a two-round RAND/UCLA Appropriateness Method (RAM) study and recruited 10 Canadian clinicians and researchers with expertise relevant to the primary care of elderly patients.

2. Line 78: Donabedian model should be defined here (in the author's own words)

Thanks for this suggestion. We have since added the bolded sentence to define the Donabedian model in the following excerpt:

Lines 78-82:	We focus on quality indicators that are directly relevant to the clinical activities of physicians (practice-based) and classified as acts of healthcare service delivery (processes). **Processes are one of three interrelated components comprising the Donabedian model – the dominant quality improvement paradigm that enables evaluations of medical care quality and the performance of health systems [21,24].**

3. Line 259: to do what?

Thanks for this comment. Based on your comment, we have re-sequenced content under the “Technical definitions” sub-heading to clarify “what was done” with the indicators/technical definitions during the panel meeting. This excerpt now better reflects how we reviewed and gathered feedback on the technical definitions in our panel group meetings:

Lines 256-327:

**Technical definitions**

From the 55 endorsed quality statements, it was necessary to develop technical definitions, an example of which is provided in S4 Table. We documented our efforts to operationalize each quality statement and posed discussion questions within the technical definition workbook used to guide our discussion in the synchronous panel meeting. For example, if the frequency of a health service encounter or timeframe for follow-up was not specified within the quality statement, we noted these outstanding aspects as points of discussion.

We referenced four ICES datasets […]

We rank-ordered the endorsed indicators from the first questionnaire to pragmatically determine a subset that would be reasonable to present in our allotted panel meeting times; it would not have been possible to discuss all 55 endorsed indicators during the predetermined two-hour timeframe. We considered what threshold of the most highly rated indicators would be feasible to present (e.g., top 10% = 6 indicators, top 25% = 14 indicators, etc.). We allotted 10 minutes to review the project objectives and facilitate introductions, 15 minutes to review ratings from the first questionnaire, and five minutes per indicator to gather feedback on the proposed technical definition – resulting in sufficient time **to review** 19 indicators (the top 35% most highly rated indicators) over 95 minutes (S5 Table; S2 Fig). […]

**Synchronous panel meeting**

We gathered feedback on the technical definitions during our synchronous panel meetings. […]

Through these discussions, we unanimously omitted four quality statements; two items were duplicates and two were not possible to accurately specify using administrative data. Panelists also suggested revising the language of eleven quality statements; these revised statements were incorporated into the second questionnaire and rated. Following the meetings, we finalized our technical definition workbook to reflect group feedback.

4. Table 2:
   --why not have 1 and 4 (vaccines) next to each other?
   --2 and 12 can both go under cognitive impairment, yes?
   --why are rows from Medical Conditions shaded and most rows from Appropriate prescribing shaded (and the others not)?

We appreciate your suggestions to improve Table 2 (Line 338). We moved indicators #1 and #4 (those pertaining to vaccines) next to each other, as you suggested. Indicators #2 (now #4) and #12 are also relevant to Cognitive Impairment, as you said; we have since added a footnote to denote this. Lastly, we regret that the shading of rows in this table was incorrect in our previous submission. No rows should have been shaded/filled; we since have corrected this.

5. Line 353: comma after available

Thanks for catching this grammatical error. We have since added a comma.

Line 412:	Measurable quality indicators are limited by information sources available, and technical definitions – which are necessary to operationalize indicators – pertain to particular settings, thereby, affecting their generalizability [21].

6. Line 384: "pragmatic reasons" is the predetermined two-hour panel meeting length, yes? If so, I would specify that to acknowledge it as a limitation "pragmatic reasons (i.e., the predetermined two-hour panel meeting length)"

Thanks for this suggestion; we have revised accordingly.

Lines 443-446:	Although excluding indicators for pragmatic reasons (i.e., the predetermined two-hour panel meeting length) may have biased our findings, our study demonstrated the utility of using a consensus process to derive a measurable indicator set for those rated most highly.

7. Figures (Figure 1, S1-2, and scatter plot figures as below) should have figure legends.

Thanks for these suggestions. We have added figure legends to Fig 1, S1 Fig, S2 Fig, and S3 Fig:

Lines 354-357:	**Fig 1. Flow diagram of endorsed indicators, by FM-COE Priority Topic**

Legend: 61 indicators across 10 FM-COE Priority Topics were identified in the literature review for rating; 55 were endorsed in the first questionnaire. Of the 19 indicators reviewed in the panel meeting, 12 indicators across four Priority Topics were endorsed in the second questionnaire.

Lines 662-673:	**S1 Fig. Literature search, organization, and screening results**

Legend: Steps 1 to 4 outline the literature search results of 282 candidate indicators. Steps 5 to 7 illustrate how the candidate indicators were organized by the FM-COE Priority Topics and streamlined into 178 quality statements. Steps 8 and 9 display the results of internal feasibility screening and clinical review, resulting in 61 quality statements for inclusion in the first questionnaire.

**S2 Fig. Distribution of summed median scores and IQRs for indicators that advanced from Round 1**

Legend: 19 indicators whose summed median scores were rated most highly were prioritized for discussion in the group panel meeting. 14 indicators had equal summed median scores and were rank-ordered by the lowest summed IQR for prioritization.

**S3 Fig. Distribution of Wilcoxon scores (matched-pairs signed-rank test)**

Legend: The p-value obtained by the Wilcoxon matched-pairs signed-rank test suggests a significant difference in consensus scores between the two RAM rounds.

8. Supplementary Figure 2:
   --x-axis: round instead of group, yes?
   --perhaps a more meaningful title than response_bias_score? Isn't this the "Wilcoxon matched-pairs signed-rank test for consensus scores"?
   --including n number for each round would be nice and clear
   --how did the Pr > Z scores translate to the p-value? wouldn't these be the same?

Thanks for these suggestions. We have incorporated the first three suggestions you provided above into S3 Fig:

In response to your fourth comment, the Wilcoxon matched-pairs signed-rank test produces two estimates (Pr > Z and Pr > |Z|) which relate to p-values. We chose to report and interpret the value 0.1303 (Pr > |Z|) in our main text, which represents the **p-value associated with the absolute value** of the Z statistic. We selected Pr > |Z| as it considers extreme values in both directions; we were not only interested in deviations in a specific direction. However, both Pr > Z and Pr > |Z| appear in S3 Fig and can be interpreted as exceeding the significance threshold of 0.05, suggesting the

null hypothesis is not rejected.

9. Supplementary Table 6: This title needs changed, correct? Items in the "Presented in panel group meeting" and "Rated in questionnaire 2" columns were prioritized in round 2, correct? The table should also be referenced accordingly in the results section, not just at the end of the discussion

Thanks for this suggestion. We have revised the title of S6 Table to be, “Prioritization of endorsed items from round 1 to round 2,” and have also referenced it in our Results on Line 311. Due to referencing this table earlier in our main text, please note that Table S6 in our previous submission is now Table S5.

10. While I acknowledge Reviewer #2's comment to reduce the number of tables/figures, I believe they are all meaningful in this revision and describe important study components. I feel the additional scatter plot figure in response to Reviewer #1 provides meaningful context to Figure 1 and Supplementary Table 6 and should be included in this submission (and would also be more "outcome focused" aligning with Reviewer #2's comments). With this though, I don't see where it was mentioned in the manuscript where 14 indicators had equal summed scores and had to be sequenced by lowest IQR. A similar scatter plot with IQR on the Y axis should thus also be included (perhaps part of the same figure as the summed median score scatter plot) for these 14 items to clearly see the raw IQR difference between the 5 included vs. other items. Were they sequenced based on summed IQR (Appropriateness + Importance)? This should be included as a result and limitation.

Thanks for affirming the importance of the additional scatter plot added in response to Reviewer #1’s previous feedback. We have since added this scatter plot as S2 Fig (Line 311). We have added a plot with summed IQRs on the y-axis to display the differences in summed IQRs for the 14 indicators with equal summed scores.

In the following excerpt from our Methods, we describe how we sequenced indicators with equal summed scores by the lowest summed IQR for appropriateness and importance:

Lines 141-146:	Among indicators deemed eligible for inclusion in the second round, we rank-ordered the indicators to develop corresponding technical definitions efficiently and feasibly. We summed the median scores for each indicator on both criteria and computed interquartile ranges (IQRs). We sequenced indicators by those with the highest summed median score. For indicators with equal summed median scores, we prioritized those with the lowest summed IQR.

In addition to including these scatterplots in S2 Fig and referring to them in our Methods, we have also added the following to our Results and Limitations:

Lines 314-316:	In round 1, 14 indicators were rated most highly with summed median scores ranging from 18 to 16.5. 14 indicators achieved the next highest summed median score of 16; from these, 5 were prioritized based on having the lowest summed IQR.

Lines 446-448:	We prioritized indicators for discussion in the panel meeting by rank-ordering endorsed indicators from round 1 with the highest summed median scores and, for those tied, the lowest summed IQR.

11. I do agree with Reviewer #3 that "operationalize" is not explicitly described. I interpret it to mean able to be examined data-wise, but I could also (I believe mis-)interpret it to mean directly used to clinical care. Can "operationalize" be described in the introduction? perhaps with the Donabedian model? or changed to "examine" if appropriate?

Thanks for this suggestion. We have since defined “operationalize” in our Introduction, as per the bolded excerpt below.

Lines 71-76:	Research focused on the development of elderly-focused quality indicators for primary care to-date have not been concerned with operationalizing them [21]. **In this context, “operationalize” refers to developing technical specifications to apply, measure, and examine indicators using available data holdings.** Despite the limitations of using secondary data to make inferences about quality [21], indicators developed without reference to information sources or those that are not feasible to operationalize within a particular context are limited in their utility and capacity to promote change.
